# 10 Limits to Forgiveness in Health Care

**DOI:** 10.1007/s10728-025-00518-1

**Published:** 2025-04-28

**Authors:** Stephen Buetow

**Affiliations:** University of Auckland, Auckland, New Zealand

## Abstract

Compliance and regulatory bodies often encourage health care providers’ disclosure of and apologies for wrongdoing. Patients may perceive that forgiveness is expected and feel pressure to grant it. However, forgiveness carries consequences, which can bring limits to forgiveness. Understanding these limits is crucial for understanding when forgiveness can either heal or add to trauma. This paper explores 10 context-dependent limits to forgiveness across four categories. The first category outlines conceptual limits: not all harm requires forgiveness, some evil acts may be beyond human forgiveness, and blame can be incompatible with forgiveness. Secondly, moral and ethical limits result from how accountability strains forgiveness, how moral absolutism can hinder it, and how proxy forgiveness may lack moral legitimacy. The third category identifies relational and social limits. Forced reconciliation can undermine forgiveness. System negligence diffuses culpability, hindering individual forgiveness, and requires prioritizing the victim’s healing and benefit despite the diluted accountability. Finally, the fourth category highlights temporal and process-related limits. It emphasizes that ongoing or unaddressed harm can obstruct forgiveness, while variations in healing trajectories may delay or complicate it. Updating current understanding, this framework adds insight into when forgiveness may be inappropriate. It offers providers ethical guidance in navigating this terrain through a person-centred approach balancing empathy and accountability. The framework aims to facilitate healing for the patient and provider, regardless of whether forgiveness occurs.

## Introduction

Sarah, a 30-year-old mother of two, dies following what should have been a routine appendectomy. The error occurred while the surgeon was working with an AI-assisted surgical robot amid system failures, including chronic understaffing, inadequate rest policies, and known software issues that hospital management had failed to address despite staff expressing concerns. The surgeon disclosed the lethal, technical error to the hospital authorities and patient’s family, which now faces pressure from the hospital to forgive the surgeon and institution.

This hypothetical case exemplifies the moral complexity of forgiveness in health care. Systems – including patient safety movements, institutional protocols, and professional culture – often require health care providers to disclose and apologize for causing harm [1, 2]. For example, in the United States, entities such as the Office of Inspector General (OIG) and Communication and Resolution Programs (CRPs) encourage providers to report improper conduct and express remorse, promoting transparency and accountability [3].

Providers who apologize may expect forgiveness despite rarely asking for it directly. However, while some cultural traditions view forgiveness as a moral duty [4, 5], forgiveness is more commonly seen as a choice [6] – a voluntary act, gifted to liberate oneself, others, or both from negative emotions [7]. This apparent freedom is constrained when victims perceive that forgiveness is anticipated and consequently feel pressure to forgive [8]. That perception can arise because the implicit social contract embedded in an apology promotes forgiveness [9] as do shared humanity [10] and power dynamics in health care relationships [11]. Nevertheless, the meaning and impact of forgiveness – defined here as an intraindividual process of consciously replacing negative feelings toward wrongdoers with either neutral or positive ones – can vary widely, with the desired endpoints of forgiveness ranging from reconciliation to detachment [12].

The effects of forgiveness are context dependent. In Sarah’s case, forgiving the surgeon who admitted erring may help each party process challenging emotions and move forward constructively [13, 14]. This potential benefit of forgiveness is particularly relevant as society grapples with the underdisclosure of errors in health care [15]. Such errors remain alarmingly common [16, 17], along with professional misconduct [18, 19]. Enabling providers to admit to and apologize for them and other professional wrongdoing may promote public health by encouraging transparency and accountability [20]. Forgiveness can also facilitate emotional healing, improve mental health for the forgiver and forgiven, strengthen provider-patient relationships, and address systemic issues to improve quality and safety in health care [21].

While forgiveness can be beneficial, recognizing its limits and inherent drawbacks is crucial. Choosing to forgive may forego the functional benefits of other cognitive and emotional responses and could increase the risk of repeated offences [22]. The freedom to withhold forgiveness challenges the notion that “there are, in principle at least, no moral limits on what we may forgive” [23]. Choosing not to forgive may preserve or limit individuals’ freedoms and opportunities. Understanding these nuances is essential to prevent instances where forgiveness may inadvertently add to the burden of harm. Such understanding should be context-dependent, accounting for factors such as the nature of the offence, the offender’s remorse and behaviour change, and the potential impact on the forgiver’s well-being and self-respect [22].

Building on these complexities in choosing to forgive or not, health care requires forgiveness boundaries. The lack of a framework for setting and applying such boundaries hinders efforts to balance accountability with compassion, especially when addressing systemic problems. Accordingly, this paper identifies 10 limits to forgiveness and discusses the implications for health care within broader philosophical and humanistic contexts. It groups the limits into four categories: conceptual limits, moral and ethical constraints, relational and social barriers, and temporal and process-related obstacles. The framework aims to support understanding of when forgiveness by patients may be unnecessary or inappropriate for quality and safety in health care.

## Conceptual Limits

Conceptual limits operate at the foundational level. Posing philosophical challenges to patient forgiveness, they question whether forgiveness is possible, appropriate, or even coherent in certain situations. Three primary constraints emerge: cases where apparent transgressions may not constitute forgivable offences, instances where the magnitude of harm transcends human capacities for forgiveness, and situations where attributing blame is incompatible with forgiving.

### Not All Harm Requires Forgiveness

Harming patients is not always sufficient for moral blame. While strict legal liability can hold providers accountable without proving fault, moral blame usually requires intent or negligence; when harm occurs unintentionally and without carelessness, moral blame is seldom assigned. Focusing on duty and intent over outcomes may remove moral culpability, potentially reducing pressure to forgive.

Critics argue that outcomes are relevant because accidents are rarely random, and they can obscure accountability for preventable mistakes [24, 25]. Moreover, forgiveness may still address any emotional or relational harm, paving the way for reconciliation. However, humans are inherently fallible [26] and calculated risks are unavoidable in fields like health care.

In Sarah’s case, the surgeon adhered to protocols to minimize risk. While remaining responsible for decision-making during the procedure, the error resulted not from carelessness but from systemic issues like insufficient rest and perhaps overreliance on technology. Blaming this physician for a mistake that could have happened to any competent professional raises a question: was moral fault (requiring forgiveness) present? However, even if not required to apologize for the technical error, the surgeon should express regret for Sarah’s family’s distress.

Forgiveness may also be unwarranted in other circumstances, such as when a higher purpose justifies some discomfort [27]. For example, a whistleblower exposing unethical practices may hurt feelings or damage reputations in the service of upholding truth and justice. A clinician correcting a patient’s harmful beliefs may cause temporary distress but aid long-term growth.

Another scenario where forgiveness is inappropriate is where the wrongdoer seeks to manipulate the *victim* into apologizing. This deceptive tactic involves perpetrators not taking responsibility for their bad behaviour. By creating doubt about moral culpability, they revictimize their targets, invalidating their negative experiences to maintain control, avoid facing repercussions, and potentially continue their harmful behaviour. The burden of reconciliation is put on the victim rather than the wrongdoer, stifling accountability and genuine healing.

Lastly, amid political correctness, the “euphemism treadmill” [28] has created a culture of apologizing for minor infractions and non-issues [29]. Such apologies can diminish their impact and meaning, appear weak, and frustrate recipients [30]. A semblance of harm may also reflect personal sensitivities rather than outright wrongdoing. What offends one person might not offend another, although knowledge of individual or cultural sensitivities may create a duty to minimize offending. Conversely, forgiveness may feel inappropriate if the offender themselves believes they have nothing that calls for forgiveness. The anger that victims may express may be merely a façade of indignation that masks other emotions like fear and insecurity.

### Evil Disallows Human Forgiveness

At the other end of the spectrum, some offences are so “radically evil” [31] that human forgiveness may be inappropriate. As Hannah Arendt [32] observed in the aftermath of World War 2, they “transcend the realm of human affairs and the potentialities of human power.” Voluntary and irreversible in violating moral principles, such offences reach a threshold of moral transgression, with or without remorse, beyond which human forgiveness is unachievable [33]. Opposing health care’s commitment to safety and wellbeing, this betrayal of fundamental trust disallows forgiveness by being incompatible with respect for self and others [34].

Although this limit to forgiveness does not apply to the surgical error in the vignette above, it is occasionally evident in health care. Systematic defilements – ranging from permanent physical and psychological traumas caused by preventable medical errors to deliberate, dehumanizing medical atrocities – reveal the potential for radical evil. Paradigmatic examples of the latter abuses include Mengele’s inhumane medical experiments at Auschwitz and Shipman’s systematic murdering of hundreds of patients in the northwest of England across decades before his eventual arrest in 1998 [35]. Such heinous acts stand out as not merely criminal but also systematic and potentially unforgivable betrayals of the sanctity of human life and the medical commitment of physicians to healing – violations that reasonably repudiate forgiveness.

Less radical but still potentially unforgivable are other severe ethical breaches. They include cases of systemic malpractice and malignant negligence that contravene basic standards of care and can inflict significant damage. Contemporary examples of irrevocable harm include the concealment of serious medical risks by pharmaceutical companies. The opioid crisis questions the possibility of forgiveness due to a systemic lack of accountability for the ongoing and preventable magnitude of suffering and loss [36]. This crisis exemplifies widespread corporate negligence and forsakes public trust, prioritizing profit over public health [37].

Derrida [38] contends that true forgiveness is of the unforgivable, while Ricoeur [39] adds that “forgiveness is directed to the unforgivable or it does not exist.” This paradox requires forgiveness to be unconditional despite and because of its seeming impossibility. Unconditionality is needed to address people’s fallibility and realize their potential for growth. However, a tension emerges: radical error precludes forgiveness by fundamentally exceeding the moral and relational boundaries within which forgiveness operates.

This dilemma raises the question of whether unconditional forgiveness is achievable, particularly when the unforgivable dismantles social or ethical frameworks to sustain forgiveness. In such instances, forgiveness risks becoming tantamount to condonation rather than serving as a personal act of liberation or moral transcendence. Extending forgiveness to radical evil threatens to erase the significance of profound transgression, collude in their immorality, and impose a heavy psychological burden on victims. Withholding forgiveness may preserve self-respect and help avoid normalizing or minimizing the gravity of contemptible acts [40].

### Blame Is Incompatible With Forgiveness

Blameworthy persons are morally culpable, deserving blame that can be incompatible with human forgiveness. Sarah’s surgeon may be blameworthy because lacerating a major artery fails to meet the expected standard of professional care. The surgeon is morally responsible for this error. As a vehicle for justice, blame emphasizes personal accountability, aims to prevent future misconduct [41], and validates the gravity of the experience of harm. Without blame, justice risks becoming symbolic rather than substantive. Yet, is blame compatible with forgiveness?

Kekes [13] argues that “when blaming wrongdoers is reasonable, there is no reason to forgive them, and when blaming them is unreasonable, there is nothing to forgive.” Forgiveness implies blame [42] and relinquishes it as a moral judgment that holds the wrongdoing against its perpetrator. Consequently, excusing or overlooking the wrongdoing through forgiveness is incompatible with justified blame and may undermine justice. The two responses to wrongdoing cannot coexist since blame sustains the moral weight of harm, while forgiveness releases it. Blame maintains moral disapproval that forgiveness inherently negates.

Critics may counter that forgiveness, without excusing wrongdoing, separates the person from the act. Disapproval of and responsibility for the act can coexist with forgiveness of the person. This perspective balances justice with compassion, enabling moral accountability and reconciliation without allowing the wrongdoing to completely define the perpetrator or relationship [43]. Offenders who take moral responsibility for their misbehaviour – such as by apologizing or making amends – are more likely to be forgiven [44]. Such forgiveness typically involves a gradual reduction in blame over time as resentment and retributive emotions diminish [45]. While prudent remembrance (rather than forgetting) of harm remains necessary to prevent future wrongs [46], genuine forgiveness transforms retributive blame into accountability without revenge, demonstrating that blame still exists – just in a non-punitive form. This reconciliation directly challenges Kekes’ claim that blame and forgiveness are incompatible.

However, Kekes might respond that urging forgiveness risks diluting the moral gravity of the wrongdoing. Forgiveness only has moral force if it replaces disapprobation by relinquishing blame and retributive emotions, such as resentment or unforgiveness. Forgiveness lacks substance and cannot genuinely occur without this emotional and moral shift.

Weil [47] expands this perspective: “The desire for vengeance is a desire for essential equilibrium. To look for the equilibrium on another level. One must go alone to the limit. There one touches the void.” Weil is claiming that the human condition allows no equilibrium and disallows forgiveness as it represents a longing to be other than human.

## Moral and Ethical Constraints

Building on the conceptual boundaries, additional moral and ethical challenges to forgiveness in health care can question its appropriateness. Three significant tensions arise: the potential conflict between maintaining professional accountability and patient forgiveness, the challenge posed by moral absolutist positions that view certain medical transgressions as unforgivable, and the ethical limitations of proxy forgiveness to achieving moral redemption.

### Accountability Strains Forgiveness

The foregoing discussion of limits alluded to a tension between accountability and forgiveness. While blame and forgiveness may seem incompatible – with blame demanding consequences and forgiveness suggesting their suspension – the relationship between provider accountability and forgiveness is more complex. Accountability serves multiple critical functions: in health care, it maintains standards through performance monitoring and peer review, enables justice when harm occurs, and drives systematic improvements to prevent future errors. Lack of justice-restoring accountability, such as through apology, limits forgiveness [44]. The challenge is to balance accountability mechanisms with opportunities for forgiveness and healing.

The South African Truth and Reconciliation Commission (TRC) exemplifies this tension. Operating from 1995 to 1998 to investigate human rights violations under apartheid, it aimed to facilitate national healing through truth-telling and conditional amnesty. However, critics argue that it “seemed to miss the point of ascribing blame for the past regime” [48]. Rooted in the ethic of *ubuntu*, its approach to forgiveness emphasized reconciliation to such a degree that it undermined accountability, offering symbolic and monetary compensation more than justice.

This historical example illuminates a crucial insight for health care: granting forgiveness too readily or broadly can erode the moral force of accountability that maintains trust and standards. Just as addressing systemic injustices in political contexts requires robust accountability mechanisms to rebuild societal trust, so must health care institutions balance forgiveness with clear accountability measures to maintain public confidence and quality of care.

For instance, in Sarah’s case, premature pressure to forgive the surgeon risks undermining accountability. While the surgeon must concede the technical error that led to her death, rushing to forgiveness before that acknowledgement and before addressing systemic failures – from chronic fatigue to unaddressed technology risks – would obstruct justice and safety improvements. The family cannot meaningfully grant forgiveness until accountability processes have identified individual and institutional responsibility, allowing them to understand what went wrong and see concrete steps implemented to prevent similar tragedies from recurring.

Without accountability and adequate corrective measures, forgiveness risks being perceived as leniency that fails to uphold justice and deter future misconduct [49]. Uncritical forgiveness risks repeating errors with impunity and compromising trust and safety in health care systems. Meanwhile, perceiving a lack of accountability, unforgiving patients may forgo necessary care.

It might be countered that, without excusing wrongdoing, forgiveness separates the person from the act, enabling both accountability and reconciliation. However, this view is problematic when it urges forgiveness at the expense of moral disapproval as a legitimate response that serves as an ethical compass for maintaining professional standards and patient safety.

### Moral Absolutism Can Be Unforgiving

In health care, a “new puritanism” [50] has developed. This social movement demands strict adherence to universal moral principles with little consideration of context or nuance. Drawing rigid lines between right and wrong, it paradoxically elevates forgiveness as an ideal while often denying it to the providers who transgress its principles. Cancel culture exemplifies this paradox.

Holding wrongdoers accountable through uncompromising moral standards, cancel culture collectively shames health care providers, among others, in the echo chambers of digital media [51]. While this approach can reinforce accountability to maintain high standards and the public trust, it risks stifling open dialogue and professional growth. It makes forgiveness harder to achieve in the pursuit of progressive norms and protecting the public interest [52] because inflexible moral standards leave little room for context, personal growth, or redemption.

Sarah’s story illustrates the potential challenges of moral absolutism in health care. Cancel culture risks publicly shaming the surgeon and denying forgiveness. Withholding forgiveness becomes a socially conditioned response that reframes error as a moral failing and seeks justice through condemnation. Forgiveness is equated with weakness or betrayal of Sarah as a victim. This environment may have unintended consequences, discouraging other providers from reporting mistakes or offering apologies, due to fear of societal demands for punitive responses.

In the 2015 gross negligence manslaughter case of Dr. Hadiza Bawa-Garba, false rumours circulated that her written reflections were used against her. The misinformation received significant attention and led other junior doctors to defensively alter their reflective practice [53]. The family did not accept her apology because the errors made were lethal and preventable.

Through a Nietzschean lens [54], unforgiveness in such circumstances represents a rejection of “slave moralit*y*,” a pragmatic refusal to relinquish power and submit in the face of harm. Cancel culture asserts a Nietzschean “master morality” that enforces its values and refuses to dilute them through forgiveness. It prioritizes justice through punishment over restoration [55], aiming to redistribute attention and resources to sidelined voices [56].

This moral absolutism, while driving institutional change, risks overlooking the systemic factors in Sarah’s case – the understaffing, inadequate rest policies, and known software issues – in favour of targeting individual accountability. Yet, the stance reflects a legitimate concern that premature forgiveness could undermine safety and advancement, particularly for vulnerable patients, weaken the deterrent effect of punishment, and potentially retraumatize those wronged.

### Proxy Forgiveness Is not Moral Redemption

Human forgiveness may be considered an illusion when granted by third parties rather than direct victims. This perspective arises from the belief that, as a deeply personal act, forgiveness requires the moral authority of the immediate targets or main causalities of the wrongdoing.

Religious teachings echo this point. For example, the Old Testament explains that only God can forgive wrongs committed against Him [57], while forgiveness for interpersonal offences belongs to those directly harmed [7]. In contrast, the New Testament recognizes God as the ultimate judge of all sins while calling for personal forgiveness on earth, as expressed in the Lord’s Prayer: “Forgive us our sins as we forgive those who sin against us.”

This principle affirms that forgiveness on earth remains within the sole purview of the victim. Third-party forgiveness oversteps these boundaries. Judaism forbids it because forgiveness is tied to the harm directly suffered by the victim. Proxy forgiveness removes personal agency that is possible, particularly when temporal distance limits longstanding injustices. Harm from collective action complicates identifying perpetrators, often in institutional contexts.

In health care, President Clinton’s federal apology for the Tuskegee Syphilis Study [58] exemplifies the challenge of addressing wrongdoing for which moral responsibility is diffuse. Such apologies symbolically and collectively acknowledge systemic harm, foster accountability, and commit to preventing future damage. Nevertheless, they may feel disloyal to and violate the autonomy of direct victims, especially when they are dead – as in Sarah’s case.

Apologies from the institution or persons other than the surgeon who erred may betray Sarah’s memory and presume her family’s moral capacity to grant forgiveness without her input. While seeking reconciliation, such proxy forgiveness – or quasi-forgiveness [59] – would at best be partial. It could even trivialize her death, impose a resolution that feels inauthentic or unwarranted, and retraumatize Sarah’s family [60]. Cultural and personal barriers further complicate third-party forgiveness. For example, Hinduism regards forgiveness as a relational and individual act, making collective apologies culturally incongruent.

Additionally, some perpetrators may not confess wrongdoing and ask for, expect, or endorse forgiveness. Whether they do or not, victims may prefer retribution [61] without clinging to anger that aggravates suffering and may degenerate into a chronic health disorder [62]. They may hold this preference when ongoing harm prompts rumination and perpetuates their grief [63]. However, their negative emotions may feel good [64]. 

As a sometimes-paradoxical experience of deriving satisfaction from resentment, holding onto justified anger in the short-term may be morally well placed in motivating victims to set boundaries with the person who harmed them [22]. While staying resentful can be self-destructive, prudently temporary anger emboldens victims to maintain interpersonal control [65], validate and protest the harm, give the wrongdoer the punishment they deserve, and sustain a sense of moral integrity and justice. Other parties may be less likely to repeat the wrongdoing out of fear of vengeful retaliation.

In health care, the risks of proxy forgiveness can become pronounced. Forgiveness of a colleague for a medical error may overstep professional standards or moral boundaries. It may undermine the professional and ethical standards that require direct acknowledgement, accountability, and remediation by the responsible party. It may also create a facade of resolution that masks structural problems, psychologically harm the victims by trivializing or bypassing their need for agency and justice, and reduce the motivation for positive social change [66].

## Relational and Social Barriers

Social dynamics and institutional structures can further impede forgiveness’s authentic expression in health care. These relational and systemic barriers extend beyond individual patient-provider interactions to encompass organizational cultures, power dynamics, and behaviour patterns. Two critical barriers emerge: institutionally pressured or coerced reconciliation, which can undermine the voluntary nature of genuine forgiveness, and systemic patterns of negligence that can overwhelm and invalidate individual acts of forgiveness.

### Forced Reconciliation Undermines Forgiveness

Demanding forgiveness is tantamount to seeking absolution on command, transforming a voluntary act into a subtle form of coercion. This pressure, often for premature emotional resolution, can overlook the complexity of victim experiences and sidestep the need for real institutional reform. By putting the responsibility to resolve harm on the harmed party, it risks further oppressing patients. Forgiveness is reduced to a transactional exchange, a tool of organizational management focused less on victim healing than preserving stability, restoring institutional image, and avoiding unwanted professional and legal repercussions.

The pressure for forgiveness imposes a moral burden on victims. This burden arises from disrespecting victims’ moral agency to withhold forgiveness and can compound over time, just as financial interest can accrue on an unpaid debt [67]. Until forgiveness is granted, the debt may exacerbate the trauma or – if victims are unwilling to forgive – their guilt for being unforgiving. This guilt may tarnish their self-image if they see forgiveness as a sign of moral weakness [68].

These problems maximize when an apology feels insincere or perfunctory. Victims may reject pseudoapologies to avoid “cheap grace” [69] since their benefits have not been earned [70]. Alternatively, they may provide role-expected forgiveness to maintain a relationship. Patients who depend on ongoing care may feel forced to forgive as a survival mechanism [51]. This desperation reflects, more generally, the power asymmetry between patients and providers. The “biopower” [71] of health care providers is evident in their medical expertise and institutional authority. Some patients already feel marginalized owing to factors like limited English proficiency, low health literacy, and disadvantaged socioeconomic status [72, 73].

Sarah’s case illustrates potential harm from forced reconciliation. Still reeling from her sudden death, her family faces pressure to forgive the surgeon and institution before they feel ready. Expecting reconciliation before fully processing their grief disregards their emotional needs and autonomy. It serves the institution’s interests in damage control and legal risk mitigation more than the family’s need for accountability and healing. It also undermines meaningful change in hospital policies and practices, perpetuating systemic harm to patients.

### System-Level Negligence Impedes Individual Forgiveness

Traditional understandings of forgiveness centre on individual culpability: an identified wrongdoer bears personal responsibility for their immoral actions and is the target of forgiveness. Systemic negligence complicates this narrative. Although mistakes take place at the front line of the health system, there is seldom malicious intent. The root causes may lie in organizational processes, policies, or cultural factors [74]. Distributed responsibility can undermine pinpointing a specific individual or group to hold accountable and forgive [75].

This diffusion of responsibility is evident in health care. Not only may the victim lack a relationship with the wrongdoing provider, but multiple providers can obscure accountability for medical errors that – as with those harming Sarah – often arise upstream. Blaming – and forgiving – her surgeon may be counterproductive amid structural failures like chronic understaffing, insufficient rest policies, and unresolved software issues. Intertwined with these design issues are impersonal breakdowns, such as inadequate communication between staff and the hospital administration. All these variables can make forgiveness psychologically challenging, especially since most erring professionals do their best in the circumstances [76].

Nevertheless, forgiveness is foremost a way for victims to move on from the emotional burden that wrongdoers foist on them and regain control of their lives [77]. While this strategy shifts the motivation of those seeking forgiveness, it also represents a constructive approach-oriented method for victims to use forgiveness as a transformative tool to help cultivate a kinder, more inclusive, and stable society conducive to forgiveness and reconciliation [78].

Despite these potential benefits, both systemic factors and legal frameworks further complicate forgiveness. Traditional legal doctrines address institutional responsibility through concepts like vicarious and tortious liability, yet these mechanisms struggle to capture the moral complexity of systemic negligence. Neither framework explicitly recognizes institutions as moral agents. While the law may treat institutions as “persons” capable of performing many actions of natural persons [79], legal personhood does not equate to the moral culpability or repentance that forgiveness presupposes. Institutions lack the moral consciousness and emotional capacity that define individual moral agency. As a result, the legal frameworks governing institutional responsibility operate on a different plane than personal forgiveness.

While institutions can take collective steps to address systemic failings, their responses may focus on structural reforms rather than genuine remorse for moral failings. Consequently, victims of systemic negligence may perceive forgiving the institution as irrelevant or counterproductive, especially if it risks validating or excusing the institutional failures that led to their harm. These issues underscore why forgiveness can be an inappropriate response to systemic negligence.

Digital technologies and online environments further diffuse individual moral responsibility for transgressions. While people are responsible for how they use technology [80], the technologies themselves can hinder forgiveness of individuals and foster digital aggression [81], These dynamics are especially evident amid persistent digital traces and context collapse, where distinct audiences merge into a single, unforgiving public [82]. Legal responses, such as the European Union’s “right to be forgotten” under the General Data Protection Regulation (GDPR), attempt to offer some protection of personal privacy but with limited and uneven success [83].

## Temporal and Process-Related Obstacles

Forgiveness in health care unfolds across time, interweaving with individual healing processes and ongoing patient-provider relationships. These temporal and process-related obstacles can limit forgiveness. Two key challenges emerge: persistent or unresolved harms can foreclose opportunities for forgiveness and individual healing trajectories can impede forgiveness. Thus, forgiveness cannot be rushed or imposed.

### Ongoing or Unaddressed Harm Can Foreclose Forgiveness

A lack of guilt or genuine remorse for causing harm that may be ongoing impedes forgiveness [84]. An inability to admit fault or apologize may reflect emotional challenges associated with acknowledging wrongdoing and losing control. In health care, the tradition of self-regulation compounds these challenges, as does fear that apologies may erode trust, indicate professional fallibility, and expose providers to professional or legal liability [85]. In the United States, state “apology laws” allow providers to apologize without the apology being used as evidence in malpractice claims, but apologies may become “empty, utilitarian, or self-serving rituals” [86].

When harm persists without being addressed, victims may feel vulnerable to ongoing exploitation. Unforgiveness then is self-protective against losing further control when forgiveness feels undeserved and might condone and perpetuate the harm. This cycle may repeat when perpetrators fail to communicate openly about what went wrong, acknowledge their transgressions, take responsibility for them, and commit to and implement positive change.

Sarah’s case indicates pressure on her family to grant forgiveness before institutional action has corrected system deficiencies. Other examples from health care include a lack of repentance by some providers for weight stigma and racial bias in pain management. Lack of remorse may sometimes stem from entrenched ideological beliefs, as in “conversion therapy” to change the gender identities of LGBTQ+ persons. Rationalization persists despite progress in apologizing for this practice [87]. Other barriers to regret include calculated attempts to deflect accountability. For instance, providers may justify substandard care by claiming mitigation from systemic pressures like understaffing, long working hours, or overwhelming caseloads.

Resource constraints, such as limited access to diagnostic tools or essential medications, can lead to rationing that disproportionately affects vulnerable populations. Similarly, the typification of specific patients as “difficult” [88] or “frustrating” [89] may result in dismissive attitudes, as seen in the neglect of patients with chronic pain conditions like fibromyalgia [90] or those exhibiting behavioural health challenges due to conditions including substance use disorders. In these contexts, demands for forgiveness may be rejected for obscuring structural accountability and perpetuating harm by normalizing inappropriate responses as unavoidable or excusable.

Historically, health care professionals and organizations have often exhibited a recurring pattern of resistance to publicly recognizing the harm caused. Examples include the Tuskegee Syphilis Study, characterized by initial denial, institutional protection, and prolonged resistance to acknowledging systemic ethical failures [91]. Proper accountability typically requires sustained external pressure followed by generational shifts and a broad societal reckoning. Inauthentic or premature forgiveness for egregious violations, such as the forced sterilizations of marginalized populations in the United States [92], may fail to break the cycle of perpetuation [91]. Instead, it risks subverting calls for forgiveness into a mechanism that tacitly sanctions medical negligence, repeating unethical behaviour without substantive consequences.

Moreover, penitence itself, even when present, can bring its own problems. They can turn forgiveness into a transaction that, Nussbaum [93] suggests, often connotes a sense of moral superiority and may require the wrongdoer to express “lowness and essential worthlessness.”

### Variation in Healing Trajectories Can Hamper Forgiveness

Another limit to forgiveness is victim unreadiness [94]. Victims have the right to heal on their own terms. Demanding forgiveness or criticizing those who withhold it is unjust because forgiveness, as a process that unfolds over time [95], can be extremely demanding for victims [96]. Victims, such as Sarah’s family, need different amounts of time and effort to process grief and consider forgiveness. Their trauma recovery is inherently variable, reflecting the personality of the individuals involved and the diversity of human resilience and vulnerability [97]. Some people, such as highly sensitive individuals [51], need more time than others to manage the negative emotions wrought by wrongdoing. This emotional work includes reflecting on values, boundaries, and relationships to obtain emotional relief and re-establish control. It avoids premature forgiveness sidestepping negative emotions as a defence mechanism, and shortcircuiting healing. Resilience is developed to reconstruct a sense of personal agency and safety.

In addition, forgiving individuals must have the cognitive and moral capacity to understand and engage with concepts like harm, apology, and reconciliation. “Forgiveness fatigue” from repeated forgiving in the context of ongoing harm can drain psychological resources and leave individuals unable to protect themselves [98]. Any support they receive for this emotional labour may be brief when required for extended periods. Thus, trajectories for forgiveness are rarely predictable or linear. They unfold as cyclical, multidimensional, and evolving experiences unique to each person. Wounds and scars may heal slowly or not at all amid lingering pain and suffering.

Externally imposed timelines for granting forgiveness can force victims to open and confront unresolved wounds or revisit past stress reactions, retraumatizing those it seeks to help. Institutionalized efforts to compress complex feelings into predetermined stages may stall personal recovery [99] and even constitute institutional or interpersonal violence, particularly when invalidating and suppressing authentic emotional experiences. Victims may feel dismissed as societal discomfort with unresolved pain prioritizes restoring social harmony over their needs and obscures systemic issues within health care. For example, institutional cultures emphasizing reconciliation over accountability may inadvertently shield providers from meaningful scrutiny.

## Moving Forward

Understanding these 10 limits to forgiveness can help health care providers better navigate harm and accountability and improve their communication about wrongdoing and resolutions to relevant parties, including patients and their families. While forgiveness can have benefits – in response or not to an apology – its appropriateness is context-dependent.

Therefore, forgiveness should not be forced but remain an organic, self-determined pathway that allows space for emotions, such as anger, grief, and mistrust [100] while empowering healing [101]. Healing depends less on forgiveness than creating a space where victims feel their suffering is acknowledged and supported in reclaiming their lives without pressure for reconciliation.

Accordingly, providers must demonstrate a commitment to fairness, acknowledge and account for harm, and promote transparency. They should avoid representing forgiveness on behalf of others. To these ends, providers can benefit from forgiveness education and training in models like the Enright Forgiveness Process Model [102]. However, they must recognize that forgiveness may not be necessary or fitting when the wrongdoing is minor or too egregious.

Significant harm calls for institutional integrity, for example, through independent investigations, possible disciplinary actions, and reparative measures over seeking forgiveness. Such measures may include compensation and systemic changes to prevent recurrence and restore dignity [93]. This approach acknowledges that forgiveness may need to occur at both the individual and institutional levels in health care. Open dialogue, facilitated by health care professionals, can help victims feel heard and supported without expecting absolution. Healing cannot be forced, and pressuring people into premature reconciliation risks undermining it.

## Conclusion

Health care providers should recognize the limits of forgiveness while learning to disclose errors and apologize effectively. This approach maintains professional accountability and upholds ethical standards without creating expectations or pressure for patients to forgive. It focuses providers on enhancing patient care and safety, reflecting on their actions, and empowering patients to process events on their own terms and timeline.

The emphasis should be on providing conditions that protect both parties from further emotional harm and can facilitate forgiveness if or when patients feel ready. Ultimately, this approach should restore trust and contribute to more just and compassionate health systems that learn from errors and prioritize the equal moral interests of patients and providers in safe, continuously improving health care. By understanding the boundaries of forgiveness, providers can navigate professional wrongdoing ethically, fostering an environment where healing – for both the patient and provider – becomes possible, regardless of whether forgiveness occurs.

## Data Availability

No datasets were generated or analysed during the current study.
